# How integrated are neurology and palliative care services? Results of a multicentre mapping exercise

**DOI:** 10.1186/s12883-016-0583-6

**Published:** 2016-05-10

**Authors:** Liesbeth M. van Vliet, Wei Gao, Daniel DiFrancesco, Vincent Crosby, Andrew Wilcock, Anthony Byrne, Ammar Al-Chalabi, K. Ray Chaudhuri, Catherine Evans, Eli Silber, Carolyn Young, Farida Malik, Rachel Quibell, Irene J. Higginson, on behalf of OPTCARE Neuro

**Affiliations:** Department of Palliative Care, Policy and Rehabilitation, Cicely Saunders Institute, King’s College London, Bessemer Road, London, SE5 9PJ UK; Nottingham University Hospitals NHS Foundation Trust, Nottingham, UK; The University of Nottingham, Nottingham, UK; Cardiff and Vale University Health Board, Cardiff, UK; Maurice Wohl Clinical Neuroscience Institute, King’s College London, Institute of Psychiatry, Psychology & Neuroscience London, London, UK; King’s College Hospital NHS Foundation Trust, London, UK; Department of Clinical Neuroscience, King’s College London, Institute of Psychiatry, Psychology & Neuroscience, London, UK; King’s College London, National Parkinson Foundation International Centre of Excellence, London, UK; Sussex Community NHS Trust, Brighton, UK; The Walton Centre NHS Foundation Trust, Liverpool, UK; St Wilfrids Hospice, Eastbourne, UK; East Sussex Healthcare NHS Trust, Eastbourne, UK; Newcastle upon Tyne Hospitals NHS Foundation Trust, Newcastle upon Tyne, UK

**Keywords:** Neurology, Palliative care, Integrated care, Intervention, Mapping, End of life care, Terminal care, Hospice, Home

## Abstract

**Background:**

Patients affected by progressive long-term neurological conditions might benefit from specialist palliative care involvement. However, little is known on how neurology and specialist palliative care services interact. This study aimed to map the current level of connections and integration between these services.

**Methods:**

The mapping exercise was conducted in eight centres with neurology and palliative care services in the United Kingdom. The data were provided by the respective neurology and specialist palliative care teams. Questions focused on: i) catchment and population served; ii) service provision and staffing; iii) integration and relationships.

**Results:**

Centres varied in size of catchment areas (39-5,840 square miles) and population served (142,000-3,500,000). Neurology and specialist palliative care were often not co-terminus. Service provisions for neurology and specialist palliative care were also varied. For example, neurology services varied in the number and type of provided clinics and palliative care services in the settings they work in. Integration was most developed in Motor Neuron Disease (MND), e.g., joint meetings were often held, followed by Parkinsonism (made up of Parkinson’s Disease (PD), Multiple-System Atrophy (MSA) and Progressive Supranuclear Palsy (PSP), with integration being more developed for MSA and PSP) and least in Multiple Sclerosis (MS), e.g., most sites had no formal links. The number of neurology patients per annum receiving specialist palliative care reflected these differences in integration (range: 9–88 MND, 3–25 Parkinsonism, and 0–5 MS).

**Conclusions:**

This mapping exercise showed heterogeneity in service provision and integration between neurology and specialist palliative care services, which varied not only between sites but also between diseases. This highlights the need and opportunities for improved models of integration, which should be rigorously tested for effectiveness.

**Electronic supplementary material:**

The online version of this article (doi:10.1186/s12883-016-0583-6) contains supplementary material, which is available to authorized users.

## Background

Patients severely affected by progressive long-term neurological conditions such as Multiple Sclerosis (MS), Parkinson’s Disease (PD), Multiple-System Atrophy (MSA), Progressive Supranuclear Palsy (PSP) (grouped together as Parkinsonism), and Motor Neuron Disease (MND) have unmet physical and psychological needs [1, 2], similar to cancer patients [3] and experience problems in coordination and continuity of care [4]. For example, Parkinsonism patients experience on average more than 10 symptoms [5], which also include many non-motor symptoms such as fatigue or psychological problems [6]. These diseases also pose significant demands on caregivers [7] who experience unmet needs [8].

Such complex, person-focused needs highlight the potential for specialist palliative care involvement. This has been recommended by the National Service Framework for long term conditions for patients with long-term neurological conditions early in the disease trajectory [9]. Palliative care aims to deliver physical, psychological, emotional and spiritual care for patients with progressive and serious illness, and their caregivers. Despite this recommendation, patients severely affected by neurological conditions often have limited access to specialist palliative care services [10].

A main factor limiting palliative care involvement in long term neurological conditions (LTNC) is the lack of evidence-based knowledge on how this should be done. So far, only a few models of integration between neurology and palliative care have been described. One Canadian palliative care service for patients with PD [11] seems to improve patients’ symptoms over 3 months [12]. However, exactly how and when patients are referred and the exact intervention format is unclear. In the UK, an integrated model in advanced MS showed improvements in patients’ symptoms, caregiver burden and costs [13, 14] after a 6-week intervention in which palliative care and neurology worked closely together. However, whether this model would also be possible and successful in other neurological conditions and settings is unclear.

To develop and test better integrated models of care between neurology and specialist palliative care for patients with several progressive long-term neurological conditions (such as MS, PD, MSA, PSP, and MND) across different care settings and services, it is needed to explore the existing type and level of integration. Such insight is still largely missing. Therefore, this study aimed to map the integration between neurology and specialist palliative care services in different centres within the UK. This is an important first step to develop and test new models of care, which could ultimately lead to better care and improved patients’ outcomes.

## Methods

### Design

A mapping exercise integrating data from a workshop, e-mail survey of centres and analysis of routine data.

### Setting

England and Wales, 8 sites purposively selected as they were larger centres with both neurology and specialist palliative care in the same catchment area and were potentially interested in taking part in a future trial of neurology and specialist palliative care. The eight sites included different geographical areas of the UK (ranging from South-East to North-West) and represented both rural and urban areas. Within the sites we had the broad range of services offered in the UK, including voluntary and NHS hospices, hospital and community based palliative care multidisciplinary teams, as well as tertiary and secondary neurological services, incorporating multidisciplinary teams for MS, Parkinsonism and MND.

### Informants

Both neurology and palliative care providers from the different centres completed the mapping document.

### Procedures

1. We hosted a workshop for the sites to explore issues that were to be subsequently considered in the mapping exercise. This identified preliminary similarities and variations and hence informed the questions asked in the mapping exercise.
2. The mapping questions were developed by IH, WG and LV with input from lead neurology and palliative care providers. The questions covered: i) the catchment area of the neurology and specialist palliative care services, population served and numbers of patients seen; ii) services and staffing; and iii) integration and relationships between neurology and specialist palliative care (e.g., joint clinics) (see Additional file 1 for the mapping questions for the neurology and specialist palliative care teams).
3. The mapping questions were sent via email to informants for each site. Key informants from all sites were contacted, who then liaised with additional members of the team to gather all information. Informants completed information via e-mail. We made use of several reminders and follow-up emails and telephone contacts in the period between June-November 2014 to obtain all information.
4. Received information was checked (by DF or LV) and if needed additional information was requested via e-mail or telephone. It sometimes proved difficult to obtain certain information, such as specific catchment areas or information on the specific number of patients seen (especially broken down to neurological conditions). Ultimately, almost all necessary information was retrieved. Some demographic information was retrieved from governmental published information, e.g., the 2011 census provided by the Office for National Statistics.^1^

### Analysis

Data were transferred into tables to facilitate comparison between sites.

## Results

### Catchment areas, population served and patients seen annually

#### Neurology

Heterogeneity in catchment area, population served and patients seen annually was found across the eight sites. Catchment areas (39-6,525 square miles) and population numbers (142,000-3,500,000) varied. MS services saw the most patients per year (<500- > 5000), followed by Parkinsonism (<150 appointments per year- > 1550 patients a year) while MND patients were seen least (10- > 400). See Table 1 for a summary of results and Additional file 2 for in-depth details for each site.

**Table 1:** Neurology and specialist palliative care service information in the eight centres in the UK

Characteristics	Condition	Neurology service	Palliative care service
Catchment area (square miles)	MS	Range: 39– 5,840	Range: 39 – 1,008
	MND	Range: 39 – 5,840	
	Parkinsonism	Range: 39 – 6,525	
Population of catchment area (number)	MS	Range: 142,000 – 3,500,000	Range: 235,000 – 1,398,000
	MND	Range: 142,000 – 3,500,000	
	Parkinsonism	Range: 300,000 – 3,500,000	
Number of patients seen annually	MS	Range: 457 – 5,018 (3510 outpatient + 1508 inpatient)	Range: 0 - 5
	MND	Range: 10 - 400	Range: 9/12 - 88
	Parkinsonism	Range: 148 (appointments per year) – 1,558 (161 inpatients, 1397 outpatients)	Range: 3 - 25
	Total – not disease specific		Range: 550 – 2,298
Service provided	MS	Frequent clinics. Some sites nurse-led telephone helplines and home visits.	Various setting; hospital, community and hospice. Most across settings
	MND	Clinics (focus on holistic, multi-professional service).	
	Parkinsonism	Clinics (inclination to work multi-professionally). Some links to community	
Integration of the two services	MS	Limited – most sites no joint visits, or formal links. Some informal relations, few sites joint clinics.	
	MND	Joined - most sites joint visits or other (formal) relationships	
	Parkinsonism	Limited to Mixed – most sites no joint visits or other formal relationships. Disease dependent – best for MSA/PSP	

#### Palliative care

Heterogeneity in catchment area, population served and patients seen annually was found across the eight sites. The catchment areas were not co-terminus (overlapping) with neurology services for six of the eight sites. Catchment areas (39- 1,008 square miles) and estimated population numbers (235,000-1,398,000) varied. Of the 550-2,298 patients that were annually seen, only a small subset were neurology patients; varying between 20 and 100. Seven of the eight sites saw less than 50 neurology patients per year (Table 1 and Additional file 2)

### Neurology service provision

Among the eight sites, the provided services (and staff) were disease specific. For MS, frequent clinics were held in almost all sites. This was sometimes supplemented by nurse-led telephone helplines or home visits. Nurses and neurology consultants made up most of the teams, often supplemented by physiotherapists and sometimes other professionals such as a psychologist (detailed data about staff not shown). For MND, services included clinics (although less often than the MS clinics) with often a focus on holistic, multi-professional service. The MND teams were multidisplinary; e.g., occupational therapists, speech and language therapists were often present, supplemented by a variety of providers such as dieticians and care coordinators. Some sites, however, only comprised of a small group, such as a consultant and nurse. Lastly, for Parkinsonism, again clinics were held at almost all sites. There also seemed to be an inclination to work multi-disciplinary (e.g., at one site there was multi-disciplinary team meeting (MDT) with input from care of the elderly) with some links to the community (e.g., telephone support or a community based nurse). However, overall in Parkinsonism the consultants and nurses formed the clinical teams. Sometimes other professionals, e.g., physiotherapist and speech therapist were involved (Table 1 & Additional file 3).

### Specialist palliative care service provision

The specialist palliative care services varied with respect to the settings they work in. Most sites worked across different settings, namely (a combination of) hospital, hospice and the community. However, some sites worked solely in one setting, e.g., hospital, community or hospice.

The staff employed at each of the sites (data not shown) reflects the multidisciplinary philosophy of palliative care, although consultants (including registrars) and (specialist) nurses formed the majority of the workforce. Social workers and occupational therapists were part of the team in a majority of sites or could be drawn upon. Other Allied Health Professionals such as a chaplain or counsellor were sometimes part of the core team. The most diverse teams were located in hospices (e.g., one site employed a creative therapist) (Table 1 and Additional file 4).

### Integration of specialist palliative care and neurology

The integration between neurology and palliative care teams varied between sites, but more clearly between diseases. For MS the integration seemed rather limited. Most sites had no joint visits or formal links. Two sites broke this trend. In one site an 8-weekly MDT meeting was held in which both MS and Palliative care teams participated while at another a 3-monthly complex problem clinic was held with Palliative Care attendance.

For MND a different picture emerged of stronger integration. Most sites had either joint clinics or Palliative Care attending MDT meetings. At one site all MND patients were invited to clinics at the hospice while at another site all patients received a palliative care assessment. Good informal links, with the MND and palliative care nurses sharing an office, was reported in one site with no joint visits. The least integrated site had no joint clinics and referrals based on needs.

Lastly, in Parkinsonism the integration seemed mixed. Around half of the sites had no joint clinics or formal relationships. Other sites had clinics or MDTs 2- to 3-monthly with one site having palliative care attendance at weekly clinics. There also seems to be a difference between the subsets of diseases. MSA and PSP patients were more often considered for palliative care input at one site, while another site hosted a PSP forum (but was not involved) and a last site had a virtual round, palliative MDT meeting and joint clinic 2-monthly for PSP/MSA patients (see Table 1 and Additional file 4).

## Discussion

This mapping exercise showed wide variation across centers in the UK regarding the neurology and specialist palliative care services available and their level of integration. The eight sites ranged in geographical location and catchment area, which was (non-linearly) reflected in the number of patients seen within each site. Exploration of the selected sites and the service provision informs understanding on the current integration between neurology and specialist palliative care, and the need and opportunities for better integrated models of care.

Overall, our results indicate that in the UK neurology patients do not often access specialist palliative care, although differences between disease groups are present. This might be problematic, as it has been estimated that between 63 and 82 % of all deaths would have needed palliative care involvement [15]. Moreover, neurology patients experience palliative care symptoms such as pain, breathlessness, worry and fatigue [3], are at the end of life regularly hospitalised [15] and it is not uncommon that they die here (quality issues with death registration data should be taken into account) [16]. Of course, neurological diseases are heterogeneous and in our sample, MS patients were seen least by specialist palliative care, followed by Parkinsonism (with more involvement for MSA and PSP then PD) and MND. This was reflected in organizational differences; the integration between specialist palliative care and neurology was least developed in MS but joint clinics or MDT meetings took place in most sites for MND patients.

Our data suggest that variations in specialist palliative care involvement are related to disease trajectory rather than disease prevalence. MND generally has a poor prognosis of a few years. This is followed by MSA and PSP, although there are subgroups in MSA such as the parkinsonian variant which may have a prognosis of 15 years. Despite the fact that PD and MS might not be fatal in themselves, they do lead to deterioration and progressive symptoms, which for MS consists of fluctuations of periods of long stability punctuated by crises, while for PD this is marked by a mixture of motor and non motor symptoms with functional consequences underpinning the advanced PD state. There are at any time 5,000 people with MND, 120,000 with PD, 3,000 with MSA, 4,000-10,000 with PSP, and 100,000 with MS in the UK [17].

There are several potential barriers, next to the unpredictable prognosis without a clear referral cut-off point, to integration between specialist palliative care and neurology. On the one hand, specialist palliative care experts have been found to be reluctant to take on care for non-cancer patients, due to a lack of disease-specific knowledge [17]. On the other hand, neurologists might not refer patients as they might not always see a role for palliative care in their (complex) patients, associate palliative care with death and dying [18], or are afraid to diminish patients’ hope by introducing palliative care [19]. Last, it could be that patients themselves might be reluctant to accept a referral, for reasons such as the association of palliative care with end-of-life care or longstanding relations with neurological services and service providers. These barriers are important to take into account and to address when developing potential models of integrated care.

Various models could explore how neurology and palliative care can complement each other so that patients may best benefit. For example, specialist palliative care could be used as an ‘add-on’ approach used in time of need. Palliative care would then be provided in addition to neurology care, without taking over. It would also imply that palliative care is provided based on need rather than on prognosis or disease stage. This reflects a shifting trend and is supported by research showing that Parkinsonism patients’ symptoms over time are better predicted by their initial level of symptoms than by their disease stage [5]. Such models fit also within the trend of a generalist and specialist palliative care approach [20], where neurology providers are taught and expected to provide primary palliative care in less complex situations. The America Stroke Association has recently endorsed such an approach for stroke patients [21]. While all providers caring for stroke patients should be able to provide primary palliative care (e.g., develop appropriate goals of care), referral to specialist palliative care should be done if necessary. While such models seem plausible, we believe they should also be rigorously tested before widely implemented.

We are currently in the process of testing such a new model within some of the sites from this mapping exercise. This exercise provided important contextual data to develop this model in line with the preparation phase of the MRC framework for developing and evaluating complex interventions [22]. Incorporating this information, our model builds further upon an earlier study showing that an integrated approach improved MS patients’ and caregivers’ outcomes, while decreasing costs [13, 14]. However, whether similar effects can be found across other diseases, managed in different settings, with varied service provisions is unclear and might become apparent from our own and future trials.

Potential factors to take into account when developing and evaluating integrated models are disease groups, co-terminus between neurology and specialist palliative care catchment areas and patients’ background characteristics. Disease groups differ, as discussed, in progression and survival. Next, the co-terminus between neurology and palliative care services could influence collaborations. If, as was the case for most sites in our sample, neurology catchment areas exceed palliative care catchment areas, neurology services need to build relationships with different palliative care teams, who might operate according to different models and which might influence integration approaches. Last, patient characteristics could influence needed care. For example, patients’ from ethnic minority groups have been found to suffer from more aggressive MS trajectories compared to white British patients [23] and are more inclined to attribute the source of their illness to supernatural powers (e.g., fate) and less to biomedical factors (e.g., genetics) [24]. They are also known to access palliative care at disproportionally lower rates [25]. These and other factors might vary between sites and might influence the most appropriate provided services or integration.

This mapping exercise had limitations. Most importantly, as we asked clinicians to provide the detailed data about not only their service provisions, but also the number of patients they see and catchment area, issues with the quality of the data emerged. Not all sites were providing all information. One of the areas that often lacked data was the population and square mileage figure. In these situations, the researchers tried to manually find them. In some cases we had to look up additional information from websites or year reports. This also served as a check for the quality of information provided, although it was not feasible to check all provided information. We were, however, impressed by the willingness from both palliative care and neurology providers to provide us with the needed information. A final limitation of this exercise was that we focused on neurology and palliative care provisions, but not on rehabilitation care (both in specialist rehabilitation centres and in the community; however in one site a rehabilitation service was involved in ongoing support of patients). This should be examined in the future, as rehabilitation can play an important role in caring for neurology patients, for example, in symptom management but also in the provision of equipment and coordination of services [26]. Other settings, such as specialist care homes, and disciplines, such as elderly care, might also play a role and be relevant to explore.

## Conclusions

Our findings show a variability in provided services and integration between neurology and specialist palliative care services, which varied not only between sites but also between diseases. This variability indicates a lack of knowledge informing which (integrated) models would work best. Therefore, we hope our findings provide an impetus for further work and development in this area to ensure neurology patients’ palliative care needs are met. New models of integrated care should be developed and tested, taking into account differences in diseases, co-terminus and demographic characteristics, with the ultimate aim of improving provided care and patients’ and caregivers’ outcomes.

### Ethical approval

No ethical approval for this study was sought as we only aimed to gather information about services, not about individual persons (or patients). Sites were anonymised in reporting; the names of sites were replaced by numbers with individual identification numbers for respective sites.

### Consent for publication

Not applicable.

## Additional files

Additional file 1: Letter and Questionnaire for Mapping Exercise. This file contains information about the mapping questions for the neurology and specialist palliative care teams involved in the mapping exercise. (DOCX 20 kb) Additional file 2: Catchment areas, population served and patients seen for neurology and palliative care services. This file contains in-depth information about the catchment areas, population served and patients seen for neurology and palliative care services, for the sites involved in the mapping exercise. (DOCX 22 kb) Additional file 3: Neurology services provided. This file contains information about the neurology services provided by the sites involved in the mapping exercise. (DOCX 17 kb) Additional file 4: Palliative care services provision and integration between palliative care and neurology. This file contains information about the palliative care services provided by the sites and the integration between palliative care and neurology services for the sites involved in the mapping exercise. (DOCX 19 kb)

## Abbreviations

LTNC: long term neurological condition
MND: motor neuron disease
MS: multiple sclerosis
MSA: multiple-system atrophy
PD: Parkinson’s disease
PSP: progressive supranuclear palsy
SIPC: short term integrated palliative care

## Footnotes

1 Office for National Statistics, 2011 Census (England and Wales). Tables accessed from: http://www.ons.gov.uk/census/2011census/2011censusdata/2011censusdatacatalogue.
