# Supplementary material for: How integrated are neurology and palliative care services? Results of a multicentre mapping exercise
Source: BMC Neurol. 2016 May 10;16:63. doi: 10.1186/s12883-016-0583-6 (PMC4862117; doi:10.1186/s12883-016-0583-6)
Supplement: Additional file 1: — Letter and Questionnaire for Mapping Exercise. This file contains information about the mapping questions for the neurology and specialist palliative care teams involved in the mapping exercise. (DOCX 20 kb) [file 12883_2016_583_MOESM1_ESM.docx]

## Additional file 1 – Letter and Questionnaire for Mapping Exercise

OPTCARE NEURO - Mapping of Palliative care services

We would be very grateful if you could answer the following questions to give us a better picture of the available palliative care services within each site and if applicable, for each disease group (PP (please make a distinction between IPD, PSP and MSA), MS and MND). This document concerns the palliative care services, a separate document will focus on the neurology services.

Your assistance in this mapping exercise will help us to achieve a better insight into the current services delivered to patients and to set up the OPTCARE Neuro intervention.

If you have any questions, please contact Dr. Liesbeth van Vliet (M: xxxx, T: xxxx)

Thank you

Dr Liesbeth van Vliet, Prof. Irene Higginson and Dr. Wei Gao

Please complete the following questions

| **Background information…** | **Please insert your answer here** |
| --- | --- |
| Name and role of person completing this exercise |  |
| Workplace (e.g. King’s College London Hospital) |  |
| Date of completion |  |

|  | **Please describe…** | **Please insert your answer here** |
| --- | --- | --- |
| 1 | The catchment area of your palliative care service (which would also provide the OPTCARE Neuro intervention - please specify if it is different for PP (if needed, please make a distinction between IPD, PSP, MSA) /MS/MND: according to: |  |
|  | a. Names of local authority boroughs covered (e.g. Southwark) |  |
|  | b. Resident population of your catchment area |  |
|  | c. Square mileage of your catchment area |  |
| 2 | What setting does your palliative care service work in (list all that apply, e.g. hospital support, community, outpatients, hospice at home, inpatient hospice) |  |
| 3 | The number of patients that are annually seen by the palliative care service, specified to: |  |
|  | a. Total number of patients |  |
|  | b. Break down for diagnosis (e.g. malignancy, neurology) |  |
|  | c. Number of patients with PP (please make a distinction between IPD, PSP, MSA) |  |
|  | d. Number of patients with MS |  |
|  | e. Number of patients with MND |  |
| 4 | The current services provided by the palliative care team (list all that apply, e.g. assessment, symptom control, organising care, liaising with other professionals, and educating other professionals), please specify if it is different for PP (if needed, please make a distinction between IPD, PSP, MSA) /MS/MND. |  |
| 5 | The systems you have for recording palliative care activity. If possible, specify this into:   1. Electronic capture 2. The specific activity data collected e.g. time to first assessment, type of holistic assessment undertaking, timing of first MDT discussion/treatment plans. |  |
| 6 | Your relation with the neurology team/services (e.g. do you have any joint clinics) per disease group: |  |
|  | a. PP (please make a distinction between IPD, PSP, MSA) |  |
|  | b. MS |  |
|  | c. MND |  |
| 7 | The staff of the palliative care team (i.e. FTE, n, which disciplines) |  |
| 8 | Any other comments you would like to add. |  |

OPTCARE NEURO - Mapping of Neurology services

We would be very grateful if you could answer the following questions to give us a better picture of the available neurology services within each site and for each disease group (PP (please make a distinction between IPD, PSP and MSA), MS and MND). This document concerns the neurology services, a separate document will focus on the palliative care services. It might be necessary that several neurologists complete this form, as they might have a specific interest in a specific disease.

Your assistance in this mapping exercise will help us to achieve a better insight into the current services delivered to patients and to set up the OPTCARE Neuro intervention.

If you have any questions, please contact Dr. Liesbeth van Vliet (M: xxx, T: xxx).

Thank you

Dr Liesbeth van Vliet, Prof. Irene Higginson and Dr. Wei Gao

Please complete the following questions

| **Background information…** | **Please insert your answer here** |
| --- | --- |
| Name and role of person completing this exercise |  |
| Workplace (e.g. King’s College London Hospital) |  |
| Date of completion |  |

|  | **Please describe…** | **Please insert your answer here** |
| --- | --- | --- |
| 1 | The catchment area of your neurology services, according to (please specify if it is different for PP (if needed, please make a distinction between IPD, PSP, MSA) /MS/MND): |  |
|  | a. Names of local authority boroughs covered (e.g. Southwark) |  |
|  | b. Resident population of your catchment area |  |
|  | c. Square miles of your catchment area |  |
| 2 | The number of patients (if needed, make a distinction between in-patients and out-patients) that are annually seen by the neurology services per disease group: |  |
|  | a. PP (please make a distinction between IPD, PSP, MSA) |  |
|  | b. MS |  |
|  | c. MND |  |
| 3 | The staff of the neurology team (i.e. FTE, n, which disciplines) per disease group: |  |
|  | a. PP (please make a distinction between IPD, PSP, MSA) |  |
|  | b. MS |  |
|  | c. MND |  |
| 4 | Your relation with the palliative care team/service (e.g. do you have any joint clinics) per disease group: |  |
|  | a. PP (please make a distinction between IPD, PSP, MSA) |  |
|  | b. MS |  |
|  | c. MND |  |
| 5 | Any other comments you would like to add. |  |
