# Supplementary material for: How integrated are neurology and palliative care services? Results of a multicentre mapping exercise
Source: BMC Neurol. 2016 May 10;16:63. doi: 10.1186/s12883-016-0583-6 (PMC4862117; doi:10.1186/s12883-016-0583-6)
Supplement: Additional file 2: — Catchment areas, population served and patients seen for neurology and palliative care services. This file contains in-depth information about the catchment areas, population served and patients seen for neurology and palliative care services, for the sites involved in the mapping exercise. (DOCX 22 kb) [file 12883_2016_583_MOESM2_ESM.docx]

Additional file 2 – Catchment areas, population served and patients seen for neurology and palliative care services

|  | 1 | 2 | 3 | 4 | 5 | 6 | 7 | 8 |
| --- | --- | --- | --- | --- | --- | --- | --- | --- |
| *Neurology* |  |  |  |  |  |  |  |  |
| Square Miles of Catchment Area | MS: 134.7  MND: 1577.2  Parkinsonism: 294.3 | MS: 5,840  MND: 5,840  Parkinsonism: 5,840 | MS: 835  MND: 835  Parkinsonism: 835 | MS: 1780  MND: 182  No data | MS: 38.55  MND: 38.55  Parkinsonism: 38.55 | *MS: 38.9*  *MND: 38.9*  Parkinsonism: *A: 38.9*  ***C:*** *451.5* | MS: 3,800  MND: 3,800  Parkinsonism: 6,525 | MS:1,008  MND:1,008  Parkinsonism: 1,008 |
| Population of Catchment Area^[[1]](#footnote-1)^ | MS: 1,712,000  MND: 3,500,000.  Parkinsonism: 3,200,000 | MS: 3,500,000  MND: 3,500,000  Parkinsonism: 3,500,000 | MS: 1,000,000  MND: 1,000,000  Parkinsonism: 1,000,000 | MS: 1,500,000  MND: 445,000  Parkinsonism: No data | MS: 300,000  MND: appr. 300,000  Parkinsonism: appr. 300,000 | *MS A: 142,000*  *MS B: 142,000*  *MND: 142,000*  Parkinsonism*: A: 142,000*  *C: 310.000* | MS: 1,600,000  MS Neurorehab: 280,000  MND: 1,600,000  Parkinsonism: 3,100,000 | MS: 1,350,000  MND: 1,000,000  Parkinsonism: 1,350,000 |
| Patients with MS seen annually | *1800* | *3510 outpatient, 1508 inpatient* | *Approx. 2,000* | Approx. 2,000 | 550 on caseload (not all active, 451 currently on list) | **A:** 57  **B:** 400 | *1600* | No data |
| Patients with MND seen annually | *400* | 270 outpatients  53 inpatients | *approximately 50* | Varied. *Approx. 32 patient’s on caseload* | 15 annually in follow up. 12-20 patients currently, 3-6 new per year | **A:** 10 | *520 clinic appts/year. 175-180 under review* | On average 12 |
| Patients with Parkinsonism seen annually | *approximately 500* | *Inpatients: 161*  *Outpatients: 1397* | *Approx. 1100 including*  *PSP and MSA: 30-50* | **No data** | 410 | **A:** 97  **C:** 660 | *148 clinic appts/year.* | 1,500 on CNS case load |
| *Palliative care* |  |  |  |  |  |  |  |  |
| Square Miles of Catchment Area | 76.73 | 180 | 334 | **A:** unknown  **B:** Inpatient unit – 183  (Community SPCT–129  Day Care – 54) | 38.55 | Approx.  330 | 44 | 1,008 |
| Population of Catchment Area | 1,398,000 | 235,000 | 665,000 | **Hospice A:** 307,000  **Hospice B:** Inpatient unit – 465,000  (Community sPCT – 220,000  Day Care - 220,000) | Approx. 300,000 | Approx. 353,600 | 260,000 | 1,350,000 |
| Patients seen annually | 1312 Referrals, 984 new referrals, 325 re-referrals | 1050 | Specialist PC Unit: Inpatient 276  Outpatient 110  Day therapy services: 102  Hospital PC Team: 1070 | **A:** 925  **B:** 1039 | 550 | 951 | Community = 769  Hospital A = 898  Hospital B= 631 | 1900 |
| Neurology patients seen annually | MS: 4  MND: 9-12  Parkinsonism: 5 | MS: 0  MND: 13  Parkinsonism: 10 | MS: 1  MND: 28  Parkinsonism: 3 *(but only mention of PSP)* | MS:5  MND: 21  Parkinsonism: 21 | MS: 1  MND: 17  Parkinsonism: 9 | MS: 3  MND: 18  Parkinsonism: 23 | MS: no data  MND: 13  Parkinsonism: 25 | MS: 3  MND: 88  Parkinsonism: 15 |
|  |  |  |  |  |  |  |  |  |

1. These data were provided by the relevant local services, or when these were not available were calculated from the populations of the cities and towns within the catchment area, as recorded in the 2011 census. [↑](#footnote-ref-1)
