# Supplementary material for: How integrated are neurology and palliative care services? Results of a multicentre mapping exercise
Source: BMC Neurol. 2016 May 10;16:63. doi: 10.1186/s12883-016-0583-6 (PMC4862117; doi:10.1186/s12883-016-0583-6)
Supplement: Additional file 3: — Neurology services provided. This file contains information about the neurology services provided by the sites involved in the mapping exercise. (DOCX 17 kb) [file 12883_2016_583_MOESM3_ESM.docx]

Additional file 3 – Neurology services provided

|  | 1 | 2 | 3 | 4 | 5 | 6 | 7 | 8 |
| --- | --- | --- | --- | --- | --- | --- | --- | --- |
|  |  |  |  |  |  |  |  |  |
| MS | Consultant runs two weekly clinics. Nurses at hospital + in community. | Multi-professional team. Consultant 6-weekly clinics and weekly MDTs meetings.  Telephone helpline.  Much collaboration with community services/non-specialists | Consultant weekly clinic. Nurse telephone advice and part of outpatient clinics. Biweekly nurse-led clinics. | Multi-professional team. Various local clinics. | 1 weekly CNS led clinics (A&B) and CNS home visits. Consultant led clinics (B).  Monthly consultant/CNS clinic (C).  Rehab services available, charity provision. Referral to other services e.g. physio. | A: provides OTs and Physios.  B: MS nurse led care | Strong CNS involvement’: clinics, Home visits/ telephone  Close relationships (e.g. OT) | Based around clinical nurse specialists |
| MND | Multidisciplinary team. Nurse deals with clinical issues, PC consultant. Clinical coordinator.  Out of hours team available. | Small multi-professional team. Fortnightly clinics (multi-professional)  Consultant-led clinics.  Telephone helpline. Assessment/intervention possible MND | Multidisciplinary team provides clinics. | Registrar clinic monthly. Care coordinator. | Consultant led weekly clinic, 5 general neuro clinic  Rehab services available, charity provision, NIV | A: OT and physiotherapist | CNS help coordinate plan and run support group.  Telephone helpline. Close collaboration (e.g. OT, diet) | Multi-professional team. CNS involved: home visits, advice, referrals. Biweekly MDM clinic District nurses provide community support.  Other services available (speech/language) |
| Parkinsonism | Multi-professional centre of expertise. Support from multi-professional team (e.g. OT) | Commitment to multi-disciplinary working. Subspecialist clinics. | Weekly consultant/ nurse clinic.  MDM input care of elderly. Nurse telephone support. Community-based nurse. | Main site in hospital: outpatient, liaising other services, satellite clinics community care of elderly. | Several weekly clinics. Home visits and in-reach to ward and hospice.  Rehab and other services (e.g. physio) are available. | A: OT and physiotherapist  C: Community visits, hospital outpatients, hospital inpatients | Consultant and nurse team. Nurse specialists run clinics + telephone helpline /links to groups in community. | One neurology clinic per week (consultant led). |
