# Supplementary material for: How integrated are neurology and palliative care services? Results of a multicentre mapping exercise
Source: BMC Neurol. 2016 May 10;16:63. doi: 10.1186/s12883-016-0583-6 (PMC4862117; doi:10.1186/s12883-016-0583-6)
Supplement: Additional file 4: — Palliative care services provision and integration between palliative care and neurology. This file contains information about the palliative care services provided by the sites and the integration between palliative care and neurology services for the sites involved in the mapping exercise. (DOCX 19 kb) [file 12883_2016_583_MOESM4_ESM.docx]

Additional file 4 – Palliative care services provision and integration between palliative care and neurology

|  | 1 | 2 | 3 | 4 | 5 | 6 | 7 | 8 |
| --- | --- | --- | --- | --- | --- | --- | --- | --- |
|  |  |  |  |  |  |  |  |  |
| Palliative care services | Hospital palliative care team, inpatients and outpatient service.  9-5 availability, Specialist Registrar weekend and bank holiday coverage | Hospital + community + hospice. Specialist PC Unit: inpatient services, outpatient, and day services, Hospice at Home, (xxx) Team (who provide non-specialist PC) | Hospital palliative care team, inpatient and outpatient, day therapy  There is also a SPC unit.  (Community PCT exclusively sees patients with cancer.) | Two hospices.  A: community, outpatient clinic, day services, inpatient unit.  B: day services, courses | The PCT works in the community, with outpatient and via Hospice at Home.  Hospice at home only nurses, community also consultant. | Hospital outpatient/  Inpatients, community, inpatient hospice, day care hospice | Two of the participating PCTs are based in hospital, and one in the community.  Hospice offers inpatient and day hospice and hospice at home | Hospice |
|  |  |  |  |  |  |  |  |  |
|  |  |  |  |  |  |  |  |  |
| *Integration between neurology and palliative care* |  |  |  |  | Outpatients at Hospice offers joint OT and physio clinic open to neuro patients, mainly attended by MND patients. |  |  |  |
|  |  |  |  |  |  |  |  |  |
| MS | Close contact. 3-monthly complex problem clinic with a PC Consultant. | No joint clinics. Referral based on needs | No formal links or joint clinics, cordial relation. Infrequent referrals | No joint clinic, referrals can be made.  A: good informal relationship  B: can liaise with CNS if required | No joint clinics. Contact on per patient basis. | A: 8 weekly MDT.  B CNS: 8 weekly MDT.  Joint visits with PC consultant if required + referral to hospice (team)  Hospice: 8weekly MDT meetings (not as strong as MND) | Ad hoc referrals. | No formal links. Occasional referral based on need. |
| MND | Joint weekly clinics.  Weekly Movement clinic which pc attends  Regular referrals  PC consultant on MND steering committee | No joint clinics. Referral based on needs | No joint clinic but MND nurse shares office with PC nurse. Much informal discussion  Macmillan nurse will see MND if requested. | Joint monthly clinic  A: Joint monthly clinic.  B: PC will soon attend MND clinics in secondary care. (MND team less involved with individual patient care following recent changes) | Monthly joined MDM meeting (plans to expand to patient clinic).  All patients at diagnosis referred to PC (mainly see OT). | A: 8 weekly MDT at hospice. Good links  Hospice: All patients MND PC assessment (following JCR) + possibility of referral to Well-being centre.  PC attends monthly MDT | PC attends monthly MDT meetings + ad hoc referrals | No joint clinics, but integrated pathway at hospice, virtual fortnightly ward round. All MND patients invited to clinic in hospice with access to PC. Specialist MND nurse in hospice |
| Parkinsonism: | Not many referrals, some interaction.  PC in weekly Movement disorder clinic + PD research group.  Less for PD, MSA/PSP are considered more for PC input (known to local PC team) | No joint clinics or relation. | No joint clinics.  Cordial relation,  infrequent referrals | Ad hoc arrangements for review by PC.  A: good informal relations  B: hosts (but not involved) in PSP forum | No joint clinics. Contact on a per patient basis | A: good links  Hospice: 8weekly MDT meetings (not as strong as MND) | PC attends 3-monthly clinics. Inpatient services are ad hoc. | Virtual round for PSP/MSA. Joint  2-montly clinic and MDT for MSA/PSP.  Other services available based on needs. Few accept referrals for PD. |
